# New methodologies for the preparation of carbon-11 labeled radiopharmaceuticals

**DOI:** 10.1007/s40336-017-0223-1

**Published:** 2017-02-25

**Authors:** Kenneth Dahl, Christer Halldin, Magnus Schou

**Affiliations:** 1 Department of Clinical Neuroscience, Centre for Psychiatric Research, Karolinska Hospital, Karolinska Institutet, 171 76 Stockholm, Sweden; 2 Department of Clinical Neuroscience, AstraZeneca Translational Science Centre, Karolinska Institutet, 171 76 Stockholm, Sweden

**Keywords:** PET, Radiochemistry, Isotopic labeling, Carbon-11, Radiopharmaceuticals

## Abstract

**Purpose:**

This short review aims to cover the more recent and promising developments of carbon-11 (^11^C) labeling radiochemistry and its utility in the production of novel radiopharmaceuticals, with special emphasis on methods that have the greatest potential to be translated for clinical positron emission tomography (PET) imaging.

**Methods:**

A survey of the literature was undertaken to identify articles focusing on methodological development in ^11^C chemistry and their use within novel radiopharmaceutical preparation. However, since ^11^C-labeling chemistry is such a narrow field of research, no systematic literature search was therefore feasible. The survey was further restricted to a specific timeframe (2000–2016) and articles in English.

**Results:**

From the literature, it is clear that the majority of ^11^C-labeled radiopharmaceuticals prepared for clinical PET studies have been radiolabeled using the standard heteroatom methylation reaction. However, a number of methodologies have been developed in recent years, both from a technical and chemical point of view. Amongst these, two protocols may have the greatest potential to be widely adapted for the preparation of ^11^C-radiopharmaceuticals in a clinical setting. First, a novel method for the direct formation of ^11^C-labeled carbonyl groups, where organic bases are utilized as [^11^C]carbon dioxide-fixation agents. The second method of clinical importance is a low-pressure ^11^C-carbonylation technique that utilizes solvable xenon gas to effectively transfer and react [^11^C]carbon monoxide in a sealed reaction vessel. Both methods appear to be general and provide simple paths to ^11^C-labeled products.

**Conclusion:**

Radiochemistry is the foundation of PET imaging which relies on the administration of a radiopharmaceutical. The demand for new radiopharmaceuticals for clinical PET imaging is increasing, and ^11^C-radiopharmaceuticals are especially important within clinical research and drug development. This review gives a comprehensive overview of the most noteworthy ^11^C-labeling methods with clinical relevance to the field of PET radiochemistry.

## Introduction

Positron emission tomography (PET) is a highly sensitive imaging modality that can provide in vivo quantitative information of biological processes at a biochemical level [1]. PET relies upon the administration of a chemical probe, often called a radiopharmaceutical, that is labeled with a short-lived positron-emitting radionuclide [e.g. ^11^C (*t*_1/2_ = 20.4 min) and ^18^F (*t*_1/2_ = 109.7 min)]. Several PET radiopharmaceuticals have been developed for imaging applications, predominantly within oncology [2] and neuroscience [3, 4]. The development of novel radiopharmaceuticals requires multiple considerations, where aspects like radionuclide selection, labeling position, metabolic stability, precursor synthesis, radiolabeling procedure, automation, quality control and regulatory affairs all have to be considered [5, 6].

Carbon-11 is one of the most useful radionuclides for PET chemistry, since its introduction into a biologically active molecule has minimal effects on the (bio)chemical properties of the compound [7, 8]. In addition, there is a vast literature on carbon-based chemistry that can be consulted in the development of radiosynthetic procedures with carbon-11. Moreover, the short half-life of ^11^C allows for longitudinal in vivo studies with repeated injections in the same subject (patient or animal) and on the same experimental day. Although the advances in ^11^C chemistry have enabled the preparation of a great number of radiolabeled molecules, there are still relatively few that have been applied for the direct preparation of novel radiopharmaceuticals for PET. The present review will provide an overview of the most recent and promising developments within carbon-11 chemistry since year 2000.

## General considerations in radiopharmaceutical chemistry

A few general comments are required to provide a context for a discussion of PET radiopharmaceutical production [2, 5]. First of all, the radionuclides used in PET emit high-energy radiation and, therefore, the traditional hands-on manipulations used in synthetic chemistry are not feasible. Thus, in order to avoid unnecessary radiation exposure, radiolabeling is performed in fully automated and pre-programmed synthesis modules housed inside lead-shielded fume hoods (hot-cells). One could say that radiochemistry, in particular that with ^11^C, is a hybrid science between organic chemistry and engineering. Time is another factor of major importance in PET chemistry. A radiopharmaceutical used in PET is typically synthesized, purified, formulated and analyzed within a timeframe of roughly 2–3 physical half-lives of the employed radionuclide. For example, to obtain ^11^C-labeled radiopharmaceutical in optimal radiochemical yield (RCY), a compromise has to be made between the chemical yield and the radioactive decay. The chemical yield of a reaction is thus not as important as the obtained radioactivity of the target compound at end of synthesis. Furthermore, since only trace amount of the radiolabeling synthon is used in PET, the amount of the non-radioactive reagents is in large excess, which implies that the reaction follows pseudo first-order kinetics. By consequence, small impurities in reagents or solvents may have a significant influence on the reaction outcome. The radiochemist has to further consider the specific activity (SA), which is a measure of the radioactivity per unit mass of the final radiolabeled compound. Since high SA is often required in neuroreceptor imaging studies to avoid saturation of the receptor system, the methods that are highlighted in this review are all non-carrier-added nature.

## Carbon-11 chemistry

**Fig. 1:**
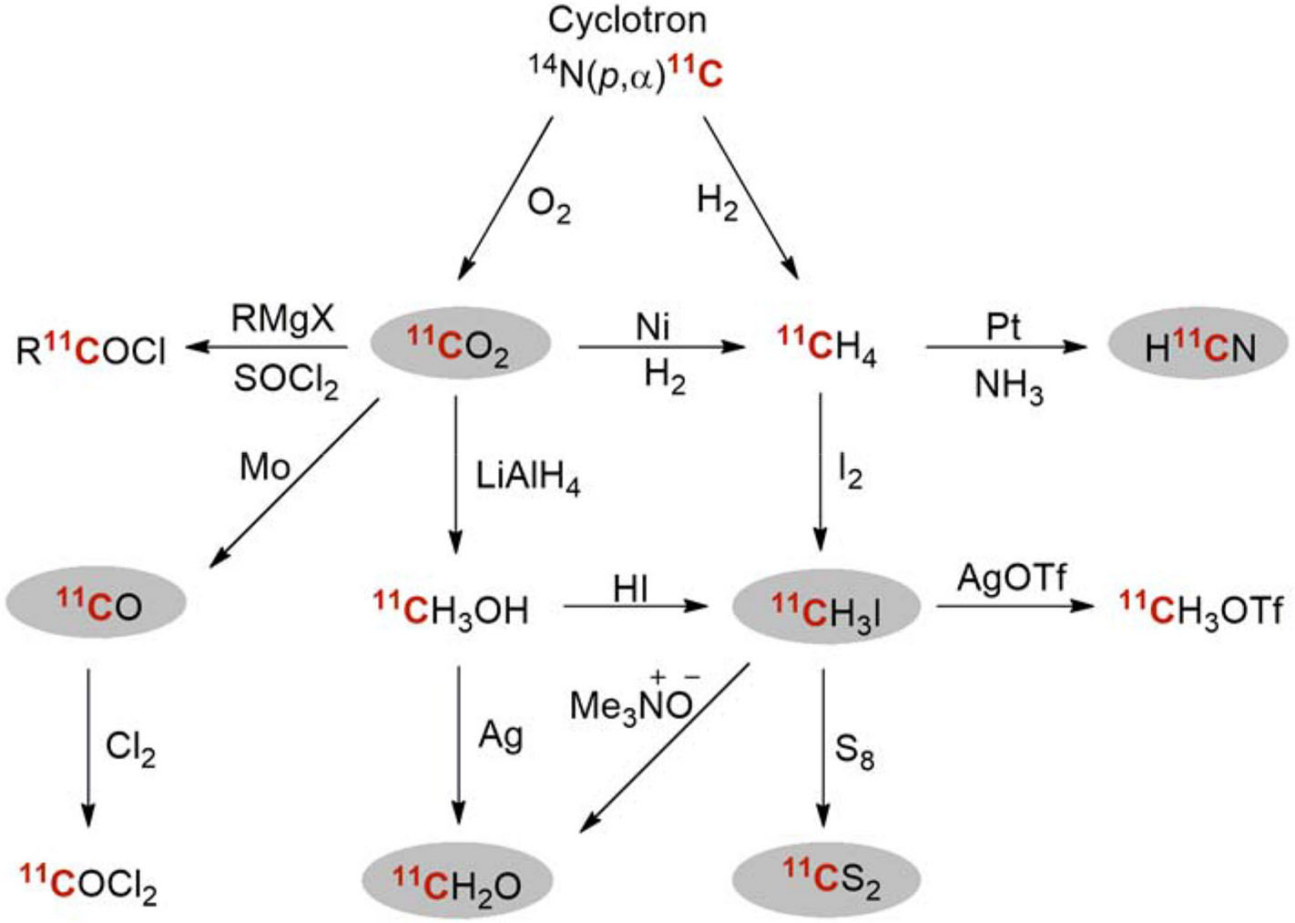
Some transformations in carbon-11 radiochemistry. Those discussed in this review are highlighted in ovals

Carbon-11 is commonly generated via the ^14^N(p, α)^11^C nuclear reaction. The reaction is performed by high-energy proton bombardment of a cyclotron target containing nitrogen gas with small amounts a second gas. [^11^C]Carbon dioxide (^11^CO_2_) and [^11^C]methane (^11^CH_4_), are formed, when either small amounts oxygen or hydrogen are present in the cyclotron target. Sometimes, these simple primary precursors are used directly as labeling agents (e.g. ^11^CO_2_), but more often they are converted via on-line synthetic pathways into more reactive species before being used in ^11^C-labeling reactions. However, several reactive ^11^C-labeled precursors have been developed over the years [8], but the ^11^C-precursors that will be discussed in this review are displayed and highlighted in Fig. 1.

## ^11^C-methylation reaction

By far, the most common method in modern carbon-11 chemistry is heteroatom methylation using the methylating agents [^11^C]methyl iodide (^11^CH_3_I) [9, 10] or [^11^C]methyl triflate (^11^CH_3_OTf) [11, 12]. This reaction can either be performed using a traditional vial-based approach or alternatively using solid support (“on-cartridge” [13] or “in-loop” [14] methods), which is very convenient from an automation prospective. A majority of the ^11^C-labeled radiopharmaceuticals that are used on a regular basis, with a few exceptions, are thus produced by these two methylating agents. However, these methylating agents are sluggishly reactive towards arylamines. Especially difficult are substrates where the aryl group in a primary arylamine electron density has been further reduced by an electron-withdrawing group. In such situations, the more reactive methylating agent, ^11^CH_3_OTf, may even fail to react. However, Pike and co-workers presented a method that utilized inorganic bases (e.g. Li_2_O) paired with DMF to permit methylation of a wide range of arylamines using ^11^CH_3_I at room temperature [15]. Moreover, in a recent study, the research group of Billard described the application of ^11^CO_2_ as a C_1_ building block for the catalytic methylation of amines [16]. Importantly, this one-pot approach eliminates the time-consuming preparation of the active methylating agent. The proposed mechanism is outlined in Table 1. In brief, an appropriate amine precursor, initially traps ^11^CO_2_ to form complex **1**, which is reduced in two-steps with ZnCl_2_/iPr and PhSiH_3_, to furnish the expected methylamine. It was realized that the ^11^CO_2_ trapping was dependent on the basicity of the amine in use, varying between 65 and 80%. A large number of substrates, including the well-established radioligand, [^11^C]PIB [17], was obtained in acceptable yields (Table 1).

**Table 1:**
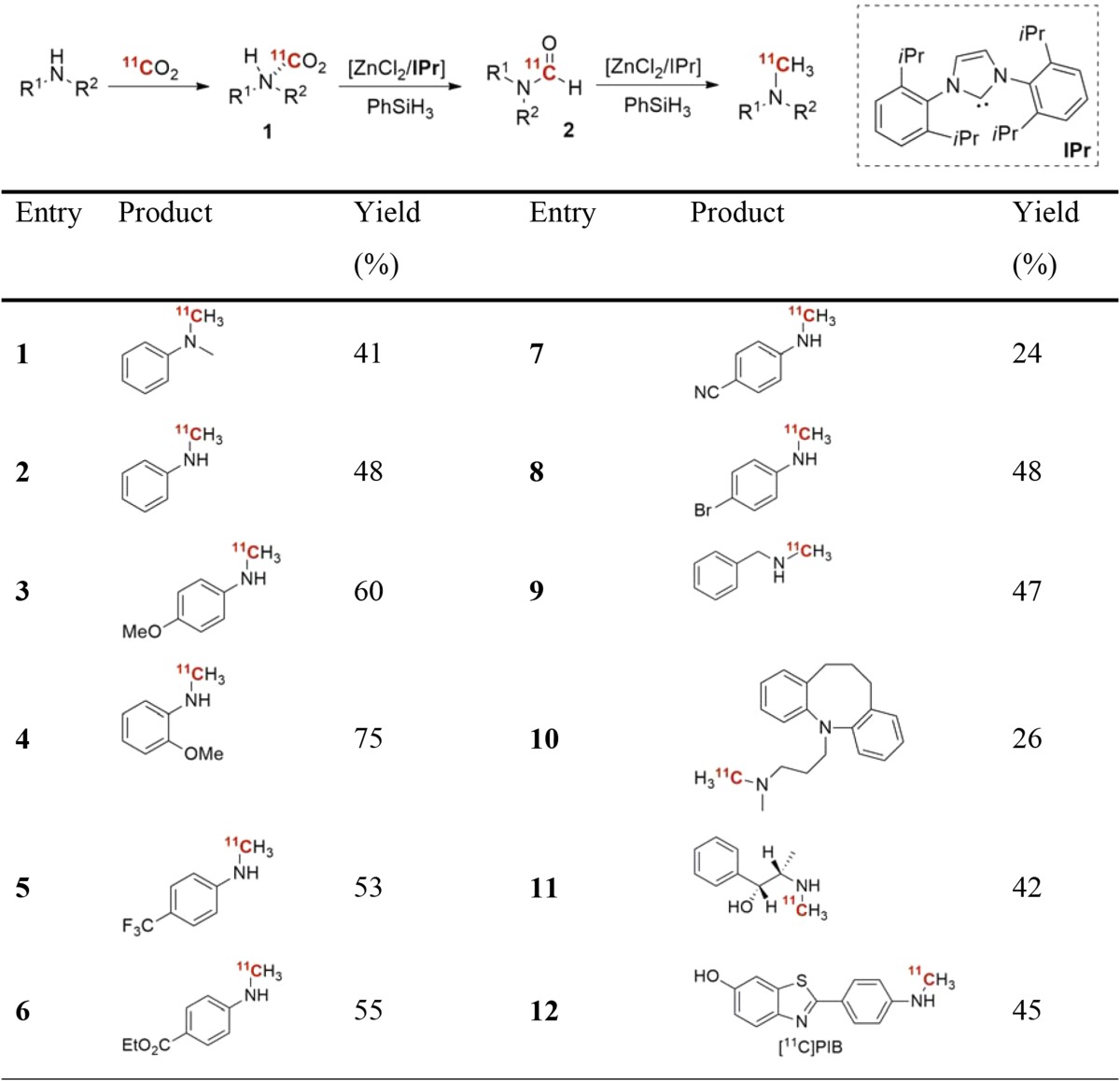
Direct ^11^C-methylation of amines using ^11^CO_2_

**Fig. 2:**
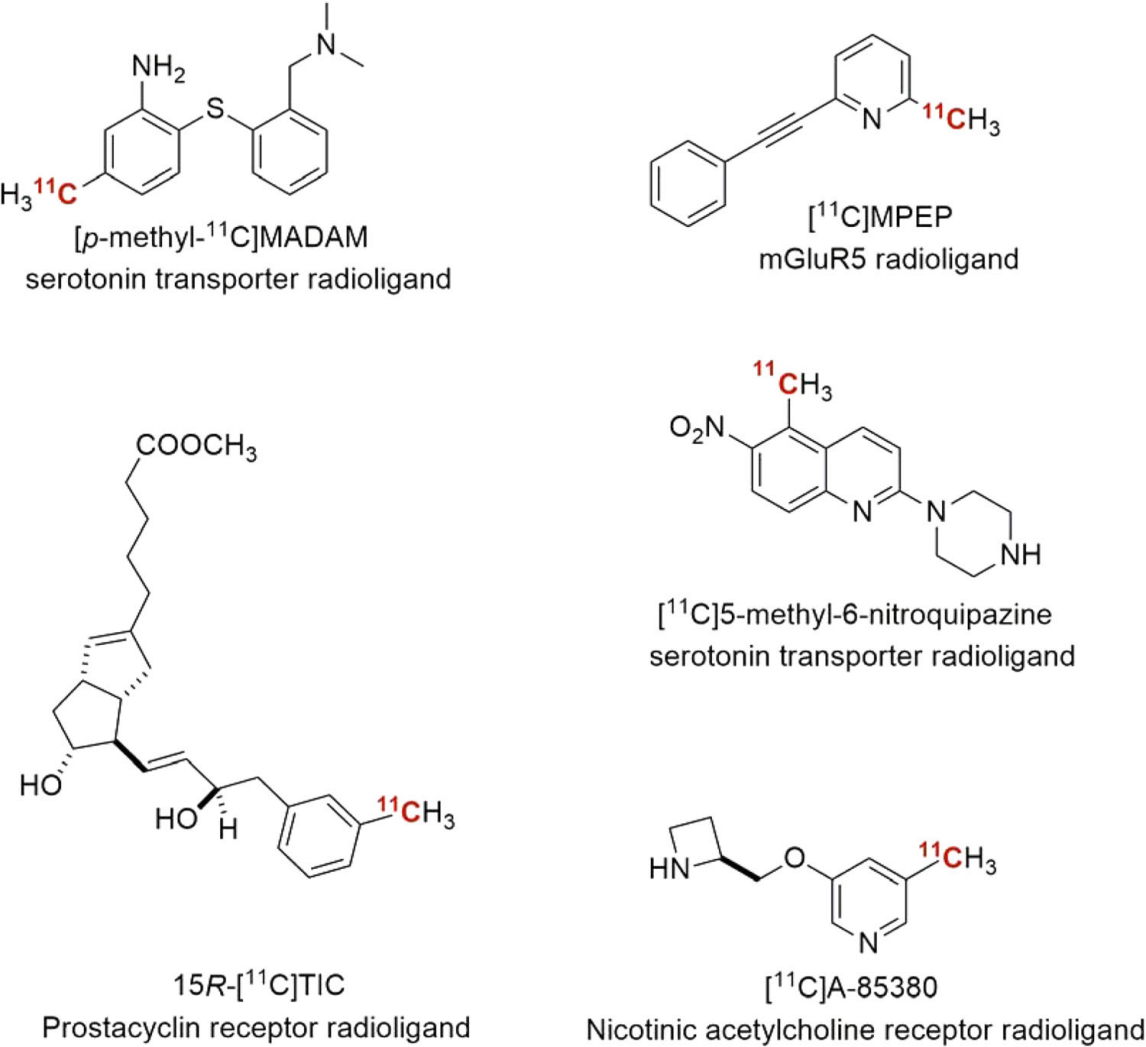
Radiopharmaceuticals labeled via metal-mediated ^11^C-methylation

In recent years, the application of ^11^CH_3_I in transition-metal-mediated reactions has become more widespread for ^11^C-labeling of radiopharmaceuticals [18, 19]. Figure 2 shows a brief overview of compounds labeled via Pd-mediated ^11^C-methylation. Two radioligands for the serotonin transporter, [*p*-methyl-^11^C]MADAM [20] and [^11^C]5-methyl-5-nitroquipazine [21], as well as a novel radioligand for the nicotinic acetylcholine receptor [22] (nAChRs) was methylated using the transition-metal-mediated reaction. [^11^C]A-85,380 displayed favorable in vivo properties for quantification of the nAChRs in living brain [23]. The nAChRs represents major neurotransmitter receptor responsible for various brain functions, and changes in the density of nAChRs have been reported in various neurodegenerative diseases, including Alzheimer’s disease and Parkinson’s disease [24]. Another example is the radiosynthesis of the 15*R*-[^11^C]TIC methyl ester, a prostacyclin receptor radioligand, which was the first radioligand approved for investigation in humans [25, 26]. The same radioligand was later used to image variations in organic anion-transporting polypeptide function in the human hepatobiliary transport system [27].

**Scheme 1:**
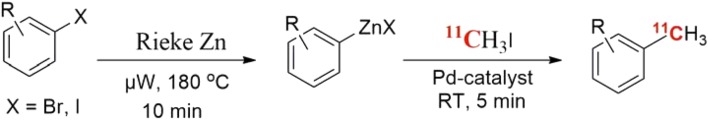
Pd-mediated cross-coupling of ^11^CH_3_I with organozinc reagent

Two of the most applied cross-coupling reactions in radiochemical synthesis today are the Stille and Suzuki reactions, where organotin and organoborane compounds function as starting materials, respectively, and ^11^CH_3_I as a coupling partner. A wide variety of functional groups such as amino, hydroxyl, or carboxylate are tolerated in these reactions and protective groups are usually not required. One unfortunate drawback with Stille coupling is, however, the inherent toxicity of the organotin reagent. Because of the regulatory aspects associated with radiopharmaceuticals that are to be used in human subjects, the less toxic organoborate substrates are usually preferred. As an alternative route to ^11^C-methylated arenes, Kealey and co-workers describe a convenient two-step Pd-mediated cross-coupling of ^11^CH_3_I with organozinc reagents (Scheme 1) [28]. The Nagishi-type reaction was used to synthesize a series of simple arenes in excellent yields. The same protocol was finally applied in the radiosynthesis of an mGluR5 radioligand, [^11^C]MPEP [29]. Even though organozinc reagents are known to be moisture sensitive, it is much likely, that in the near future, Nagishi cross-coupling reaction will be considered a good alternative to the established protocols.

**Scheme 2:**
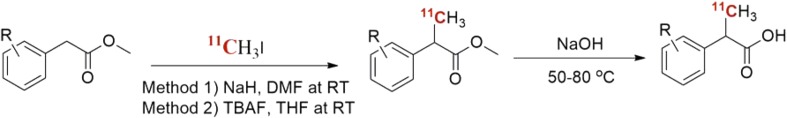
Synthesis of ^11^C-labeled 2-arylpropionic acids and their methyl esters

Enolates are a class of carbon centered nucleophiles that shortly may have a major importance in the radiopharmaceutical community. To generate an active enolate, a strong base, such as alkyl lithium or lithium diisopropylamide is typically needed. Using lithium bases to remove a α-proton is not always adequate because of their moisture sensitivity. However, in 2010, two methods for the synthesis of ^11^C-labeled arylpropionic acid derivatives have been presented [30, 31]. The rapid sp^3^–sp^3^ ^11^C-methylation reaction relied on the formation of benzylic enolates, using either sodium hydride or tetrabutylammonium fluoride as base (Scheme 2). The reaction proceeds smoothly under mild conditions. However, until recently, the ^11^C-methylated product formed under these conditions was obtained in low enantiomeric purity. The use of chiral phase-transfer catalyst has enabled enantioselective synthesis of the amino acid, [^11^C]l-alanine, in high enantioselective purity [enantiomeric excess (ee) of 90%] [32].

**Scheme 3:**
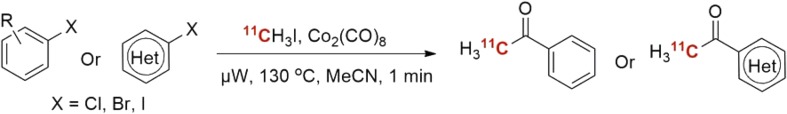
The formation of aryl methyl ketones via direct ^11^C-acetylation with ^11^CH_3_I

Moreover, this year, our group presented a novel (carbonyl)cobalt-mediated, and microwave-assisted, carbonylative protocol for the direct preparation of ^11^C-labeled aryl methyl ketones using ^11^CH_3_I as the labeling agent [33]. The method uses CO_2_(CO)_8_ as a combined aryl halide activator and carbon monoxide source for the carbonylation reaction. The method was used to label a set of functionalized (hetero)arenes with yields ranging from 22 to 63% (Scheme 3).

## ^11^CO_2_-fixation reaction

**Scheme 4:**
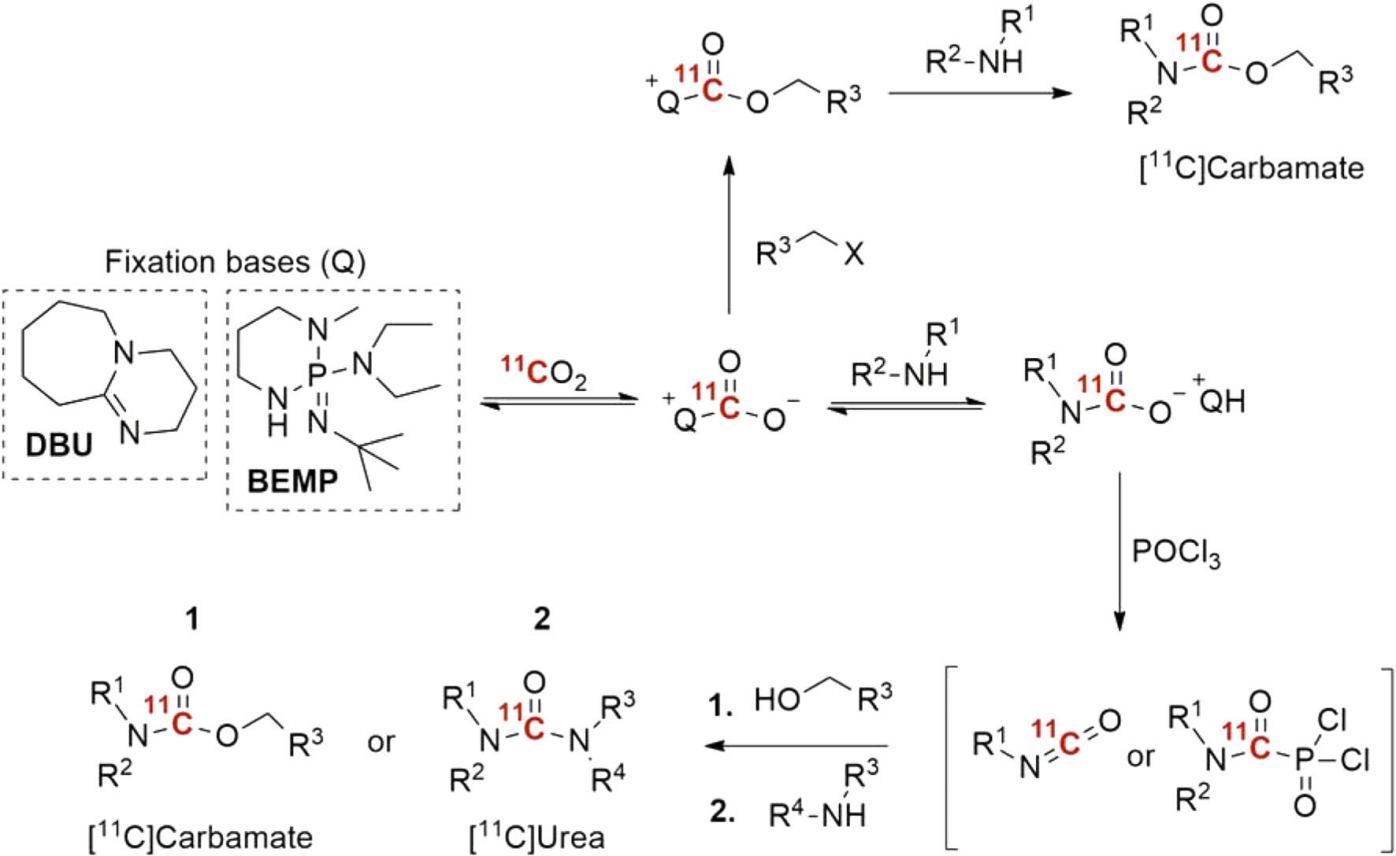
Proposed pathways of ^11^C-labeled urea and carbamates via ^11^CO_2_-fixation chemistry [37]

**Fig. 3:**
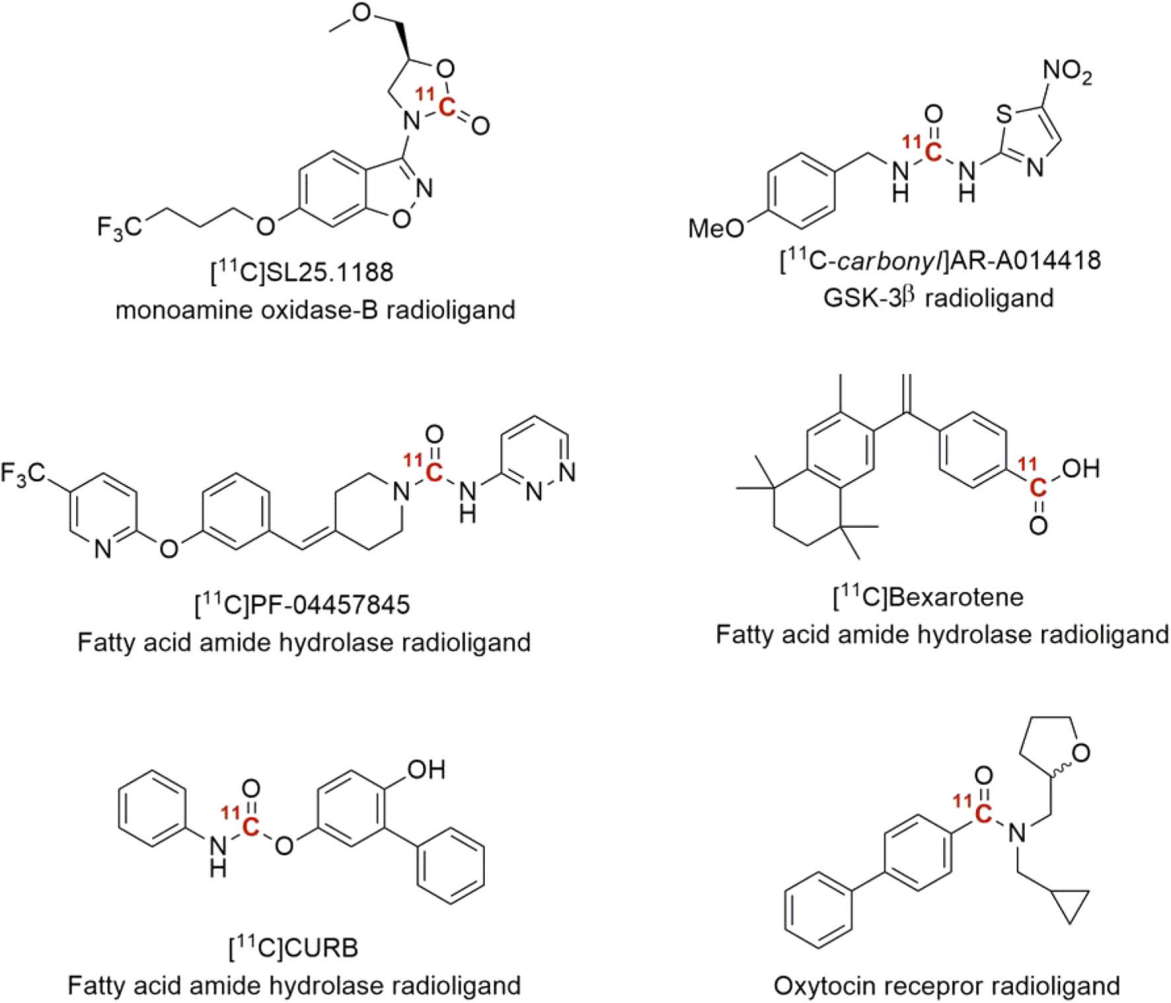
Radiopharmaceuticals labeled via ^11^CO_2_-fixation chemistry

[^11^C]Carbon dioxide is in itself a highly attractive starting material for radiolabeling, since it is produced directly in the cyclotron. However, due to low chemical reactivity, the direct incorporation of CO_2_ into organic molecules poses a significant challenge. High pressures, high temperatures or catalysts are commonly required to activate the molecule. The traditional method for ^11^CO_2_ “fixation” is the Grignard reaction, which involves the conversion of alkyl or aryl magnesium halides to [^11^C]carboxylic acids. However, Grignard reagents require great care and the rigorous exclusion of atmospheric moisture and CO_2_ during storage and manipulation. To overcome these limitations, two independent research groups presented what arguably can be viewed as the most ground-breaking advance in the field of carbon-11 chemistry since ^11^CH_3_I was introduced in the early 1970s. The innovative method, that was inspired by the recent advances in “green chemistry” and reported in 2009, uses sub-milligram amounts of precursor compound, reacts at low temperature (typically room temperature), for 1–3 min reaction time and does not require advanced technical equipment [34, 35]. To overcome the low reactivity of CO_2_, organic amines such as DBU or BEMP act as organomediators by activating CO_2_ prior to the covalent bond formation [36, 37]. The first report on ^11^CO_2_ fixation was on the synthesis of ^11^C-labeled carbamates. However, the scope of the method was later broadened to include [^11^C]ureas and [^11^C]oxazolidinones (Scheme 4) [38], via the formation of an ^11^C-labeled isocyanate or carbamoyl anhydride intermediate. A number of drug-like molecules have been prepared using this methodology in recent years (2009–2016, see Fig. 3). These includes, the carbonyl analogue radioligand of [^11^C-methyl]AR-A014418, a compound developed for imaging of synthase kinase 3β (GSK-3β) [39]. However, unfortunately, the in vivo evaluations of AR-A014418 revealed an undesirably low brain uptake [40]. Moreover, two potent and irreversible fatty acid amide hydrolase (FAAH) inhibitors, [^11^C]PF-04457845 [41] and [^11^C]CURB [42], have also been prepared. The latter, [^11^C]CURB, have recently been translated to a clinical setting for reginal quantification of FAAH activity in human brain [43]. Furthermore, the reversible monoamine oxidase B (MAO-B) radioligand, [^11^C]SL25.1188, previously prepared using the technical demanding [^11^C]phosgene approach, was radiolabeled in high yield via ^11^CO_2_-fixation [44, 45]. This radioligand was recently translated for human PET imaging [46].

**Scheme 5:**
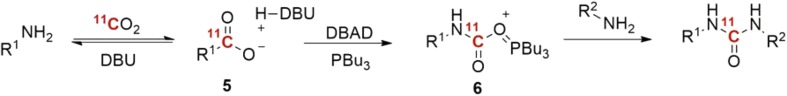
Proposed pathway for ^11^C-labeled urea formation using Mitsunobu reagents

Later, on a related subject, Dheere and co-workers presented a further refinement to the methodology to obtain [^11^C]ureas from less reactive amines, such as anilines [47, 48]. Once again, DBU was used to trap ^11^CO_2_ in solution, but in this case, it was shown that treatment of the carbamate anion intermediate (**5**) with Mitsunobu reagents, DBAD and PBu_3_, provided the corresponding asymmetric ureas in high radiochemical conversion (Scheme 5).

In the interest of expanding the scope of ^11^CO_2_ as a feedstock in radiochemical synthesis, copper-mediated approaches to carboxylic acids and their derivatives have been described [49, 50]. In the most recent example, the combination of Cu(I) with boronic esters enabled CO_2_ activation in the presence of a soluble fluoride additive and an organic base. In this reaction, the use of TMEDA was found to be crucial for obtaining high radiochemical yields, an observation likely explained by its dual action as both a trapping agent for ^11^CO_2_ and a ligand for the copper catalyst. A variety of functional groups were tolerated under optimized conditions, and the generated ^11^C-carboxylic acids could be further converted into either amines or esters, as exemplified in the one-pot two-step preparation of a candidate radioligand for the oxytocin receptor.

## Carbonylation reactions using ^11^CO

[^11^C]Carbon monoxide (^11^CO) has many attractive features as a synthon for PET radiochemistry, including its facile production [51, 52] and high versatility in transition-metal-mediated carbonylation reactions [53–56]. The widespread use of ^11^CO in radiosynthetic chemistry was until recently hampered by its poor reactivity. Several solutions have been introduced to overcome the above shortcomings, both from a technical and chemical point of view [57–60]. A breakthrough was reported in 1999, where Kihlberg and co-workers introduced a method wherein ^11^CO was allowed to react in a small autoclave under high solvent pressure (>350 Bar) [61]. The high-pressure reactor methodology exhibited nearly quantitative ^11^CO trapping efficiency and high radiochemical yield. Even though this method has exemplified the importance of ^11^CO as a labeling precursor it has not gained broad adoption in the PET radiochemistry community. This can partly be attributed to the overall complexity of the autoclave system and the relatively high level of service needed to maintain the system operational. Moreover, the repeated use of an integrated stainless steel reactor may infer issues related to transition metal build up over time, which is problematic in reaction development and system validation.

**Fig. 4:**
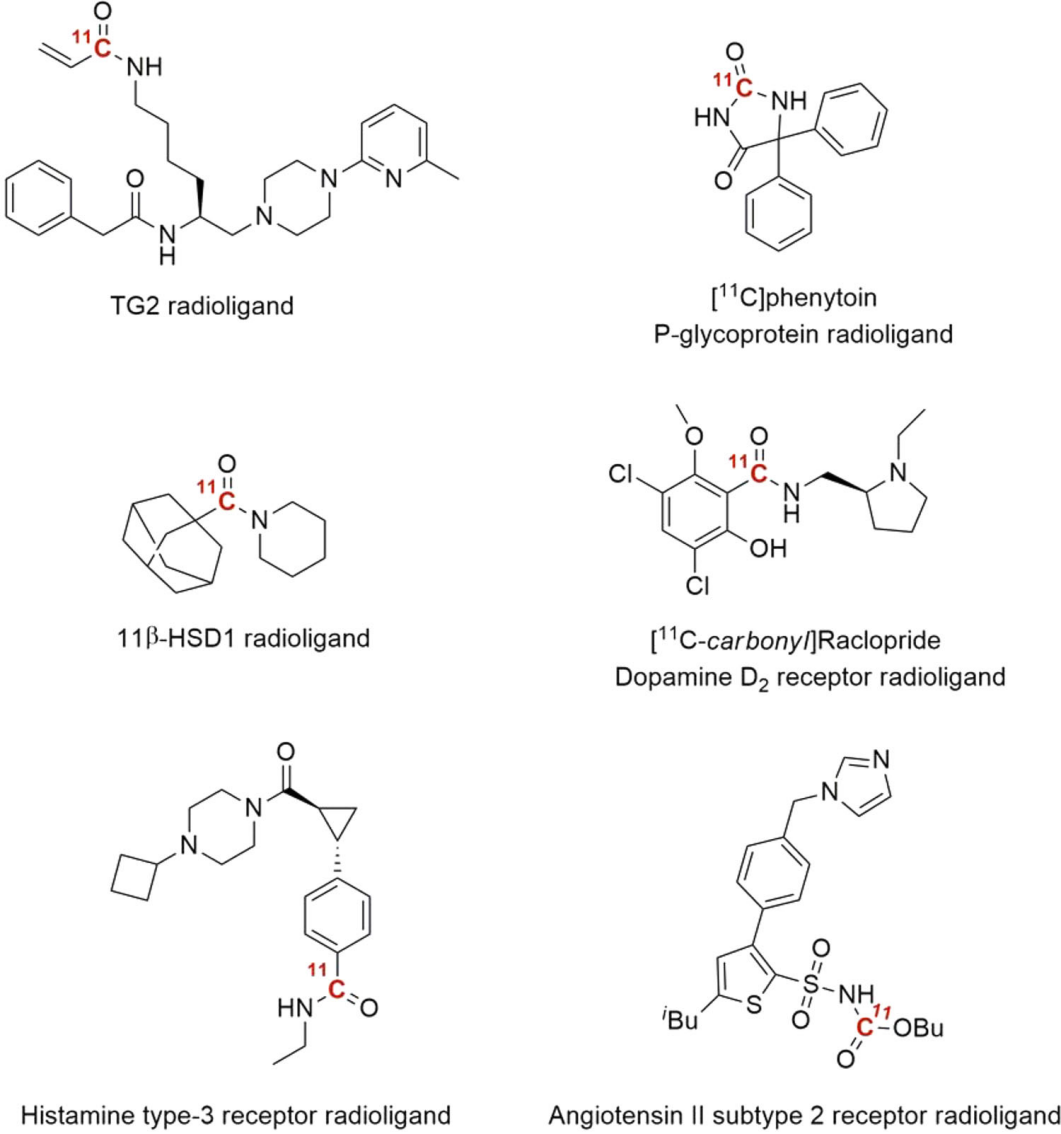
Radiopharmaceuticals labeled via ^11^CO low-pressure techniques

In recent years, the development of low-pressure techniques has been in focus. In 2012, an efficient protocol was reported by Eriksson and co-workers, in which ^11^C-carbonylation reactions were achieved without the need for high-pressure equipment [62]. The high solubility of xenon gas in organic solvents was exploited as an effective way of transferring ^11^CO into a sealed standard disposable reaction vial (1 ml) without significant pressure increase. The utility of the method was exemplified by ^11^C-labeling of amides, ureas, and esters. The use of disposable glass reaction vessels eliminates carry over issues associated with the high-pressure autoclave system, thus simplifying the transition to clinical applications. Recently, three reports were published using the same ^11^CO transfer protocol (Fig. 4). Windhorst et al. produced three ^11^C-labeled acryl amide radioligands for in vivo PET imaging of the tissue transglutaminase (TG2) enzyme [63]. Moreover, with the “xenon-method” as the synthesis platform, the Uppsala-group presented two novel approaches to ^11^C-carbonyl labeled compounds. Firstly, a new multicomponent reaction for ^11^C-labeling of sulfonyl carbamates was described [64]. The method was further applied as a synthetic tool for the in vivo evaluation of an angiotensin II receptor subtype 2 (AT_2_R) agonist. Secondly, a method to access ^11^C-labeled alkyl amides via a thermally-initiated radical reductive dehalogenative approach [65]. One of the restrictions of transition-metal-mediated reactions is the competing β-hydride elimination of the resulting metal-substrate intermediate, which precludes the use of alkyl electrophiles containing β-hydrogen. This unfortunate competing reaction is non-excitant for radical pathways. A series of un-activated alkyl iodides was successfully converted into the corresponding alkyl amide in good RCY, including the radiosynthesis of an 11β-HSD1 inhibitor.

Specific Pd-complexes have also been shown to trap ^11^CO at ambient pressure and without the need for any high-pressure equipment [66, 67]. XantPhos, a hindered bidentate phosphine ligand, in combination with palladium (µ-cinnamyl) chloride dimer were found to be excellent for promoting ^11^C-carbonylation reactions. Notably, in this study it was discovered that, depending on the palladium-ligand complex in use, different ^11^CO trapping efficiency was observed (Table 2). The reaction proceeds smoothly at close to atmospheric pressure with aryl halides or triflates as substrates using simple disposable glass vials. This method was recently also applied in the preparation of well-known D_2_ radioligand, [^11^C]raclopride, but with the carbon-11 labeled in the more metabolically stable carbonyl group (Fig. 4) [68]. Interestingly, in a direct comparison between ([^11^C]*methyl*)raclopride (produced using the standard ^11^C-methylation approach) and ([^11^C]*carbonyl*)raclopride, both radioligands showed similar in vivo properties with regards to quantitative outcome measurements, radiometabolite formation and protein binding.

**Table 2:**
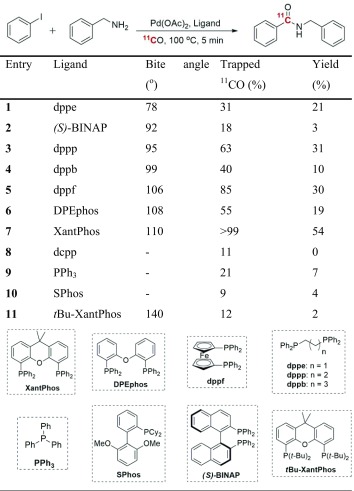
Ligand effect in ^11^C-aminocarbonylation reaction at ambient pressure

**Scheme 6:**
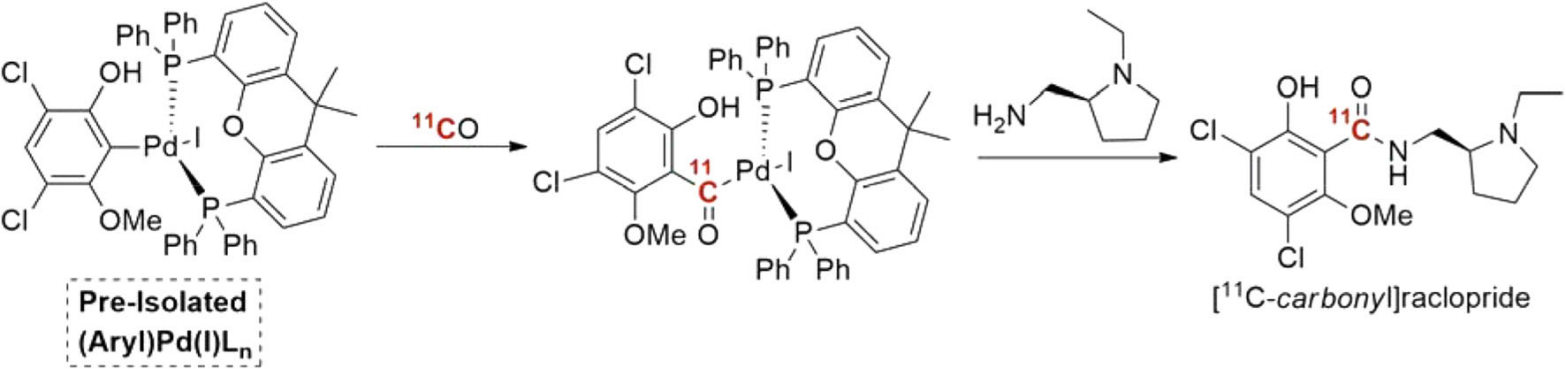
Labeling based on pre-isolated (Aryl)Pd(I)L_n_ complexes with ^11^CO

The protocol was further improved by Andersen and co-workers, where pre-generated (Aryl)Pd(I)L_n_ oxidative addition complexes were utilized as precursors for the following ^11^C-carbonylation reaction [69]. This is exemplified in the preparation of [^11^C- *carbonyl*]raclopride in Scheme 6. The isolated complexes, (Aryl)Pd(I)L_n_, have already undergone the potentially challenging oxidative addition step before their employment in carbonylative ^11^C-labeling. In this case, Pd-XantPhos complexes appeared to be among the most reactive precursors, although, electron-deficient aryl precursors demanded Pd-P(*t*-Bu)_3_ to prevent aryl scrambling with phosphine ligand. The simplicity of these low-pressure techniques, and especially the “xenon-method” delivery protocol, may offer a potential for being widely adopted in radiopharmaceutical research and development.

As mentioned previously, one restriction with transition-metal-mediated reactions is the competing β-hydride elimination. However, in contrast to Pd or Rh catalyst, nickel has been known to suppress the β-hydride elimination reaction. In the light of this, Rahman and co-workers recently reported the first successful use of nickel-mediated carbonylative cross-coupling of non-activated alkyl iodides using ^11^CO at ambient pressure [70]. The best conditions identified in this study made use of a nickel(0) precatalyst, Ni(COD)_2_, in the presence of bathophenantroline as ligand (Table 3). Six model compounds were successfully radiolabeled in acceptable to good yields. However, more data is required to establish if the method is suitable of preparing more complex molecules.

**Table 3:**
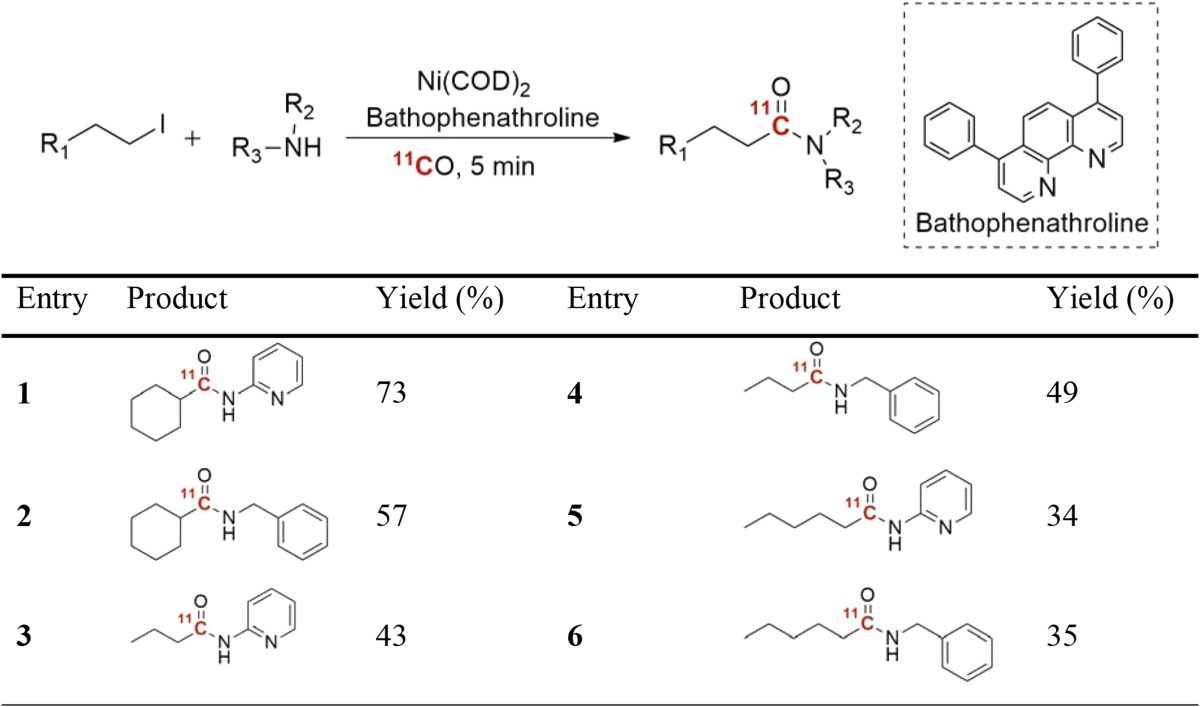
Nickel-mediated ^11^C-aminocarbonylation of iodoalkyl compounds

## Other recent advances in carbon-11 chemistry

**Fig. 5:**
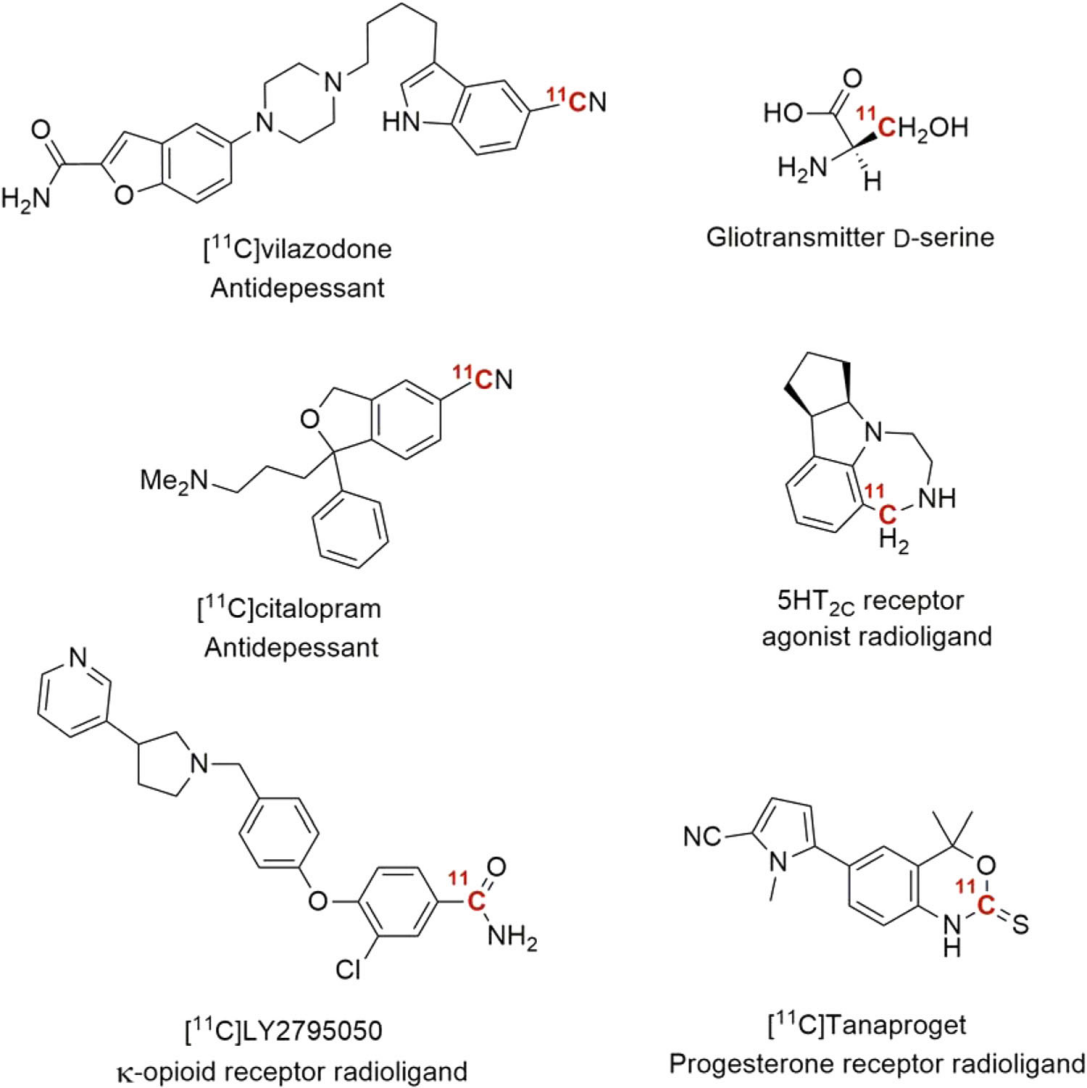
Radiopharmaceuticals labeled using either ^11^CN, ^11^CS_2_ and ^11^CH_2_O

Hydrogen cyanide is well established as a versatile precursor in PET radiopharmaceutical chemistry [71–73], and its involvement in metal-mediated cyanation of aryl (pseudo)halides is well documented [74, 75]. A limitation of such reactions is that they require rather harsh conditions, such as high temperature, long reaction times, and inorganic bases (e.g. KOH), which reduces the substrate scope. Recently, a novel method was reported describing near instantaneous, room temperature Pd-mediated coupling of [^11^C]HCN to aryl halides or triflates [76]. The method is based on sterically hindered biaryl phosphine ligands (Table 4) that facilitate rapid transmetalation with [^11^C]HCN and reductive elimination of aryl nitriles at ambient temperature. A wide variety of (hetero)arenes and drug-like molecules were radiolabeled in high yields, including the κ-opioid receptor radioligand [^11^C]LY2795050 (Fig. 5). Moreover, two known antidepressants were also ^11^C-labeled in this study. This further illustrates the usefulness of the current method in the preparation of radiopharmaceuticals.

**Table 4:**
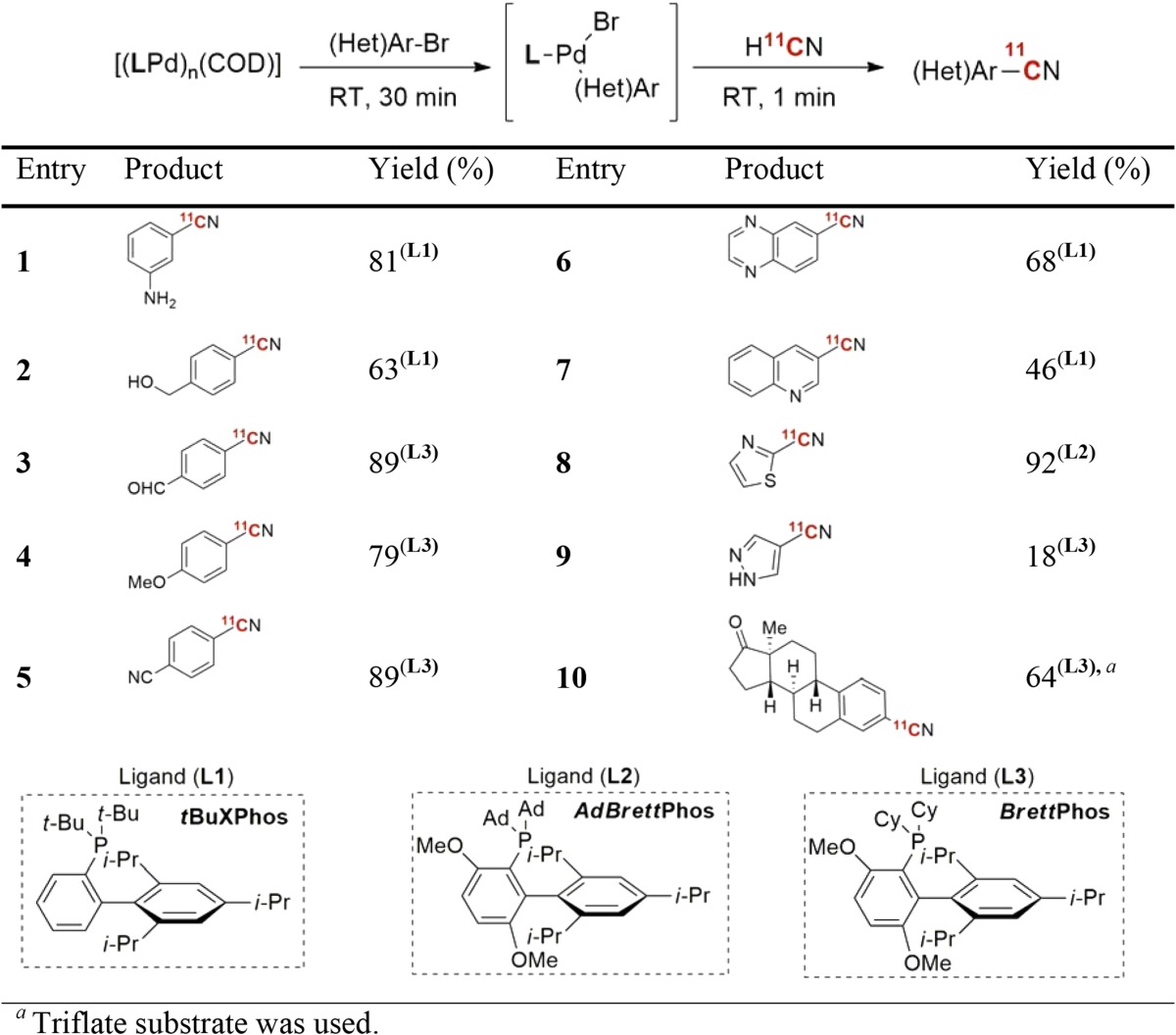
Pd-mediated radiosynthesis of [^11^C]aryl nitriles

**Scheme 7:**
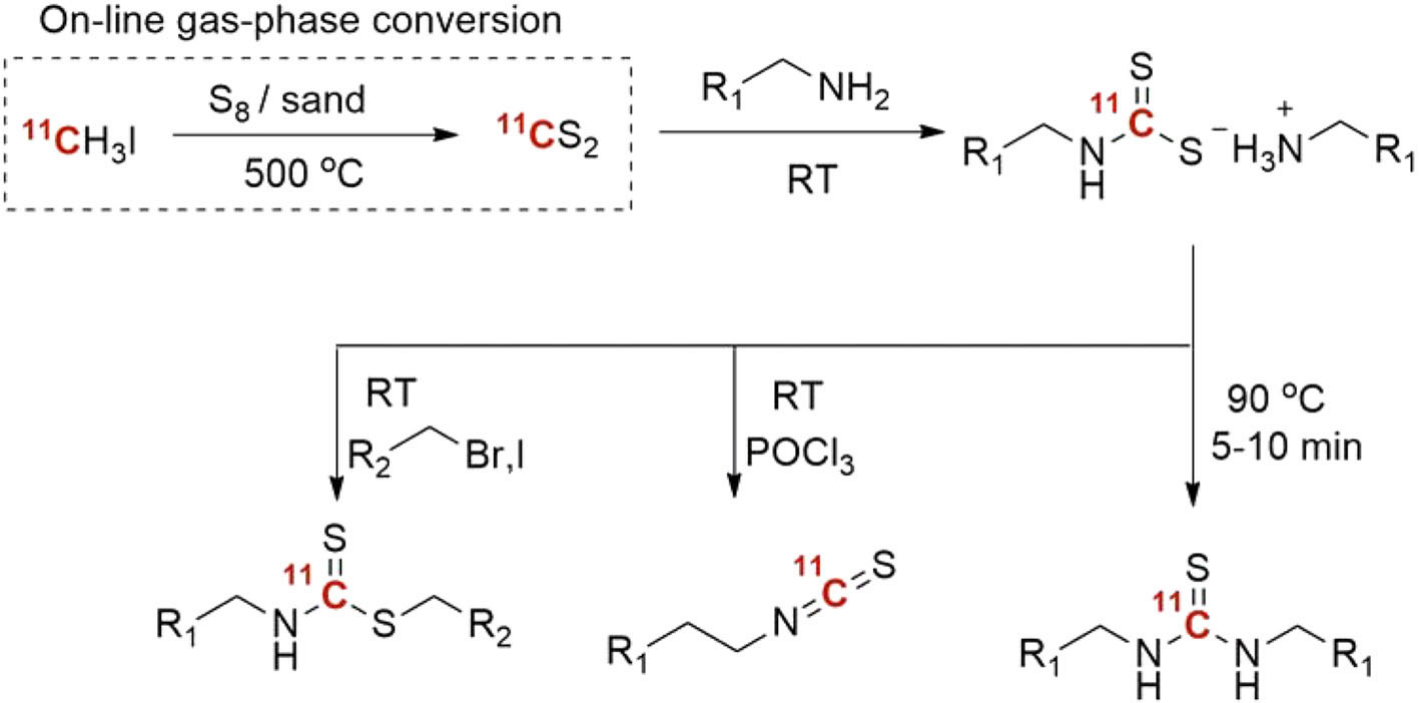
^11^CS_2_-fixation to form thioureas, thiocarbamates and thioisocyanate

Carbon disulfide, the sulfur analogue of carbon dioxide, has recently been synthesized on-line from ^11^CH_3_I using either P_2_S_5_ and elemental sulfur (S_8_) at elevated temperatures in excellent yields [77, 78]. Due to the weaker C=S bond CS_2_ is considered more reactive than CO_2_. Moreover, CS_2_ reacts very rapidly with many primary amines at room temperature to form the dithiocarbamate salts, which in turn, upon treatment with a suitable alkylating reagent will give the corresponding thiocarbamates. Heating, on the other hand, induces rearrangement to form the symmetrical thiourea (Scheme 7). Some model compounds were radiolabeled using this protocol in near quantitative yields. Finally, a progesterone receptor agonist, Tanaproget, was also produced in high RCY (Fig. 5).

Lastly, an improved, mild synthesis of [^11^C]formaldehyde have opened up new to carbon-11 labeled radiopharmaceuticals [79]. The treatment of trimethylamine *N*-oxide with ^11^CH_3_I at room temperature gave ^11^CH_2_O in a one-pot reaction. This novel preparation has been utilized by a number of groups to generate new exciting compounds (Fig. 5) [80, 81]. In addition, since [^11^C]formaldehyde was reported already in 1972 [82], there are other molecules previously reported in the literature that may now be synthesized in a simplified fashion using this protocol.

## Final remarks

The increasing importance of PET in drug development and clinical research has motivated researchers to initiate programs directly dedicated to development of new radiolabeling methods. This review summarizes some of the most recent and promising strategies to obtain carbon-11 labeled products. In the past two decades, and well before this, efforts have brought to bear an impressive range of methods for ^11^C-radiochemistry. However, there are still issues to be addressed. Take for example, the heteroatom ^11^C-methylation reaction, which is now considered as an established method by the broader radiochemical community. Why is this? The main reason is the access to dedicated commercially available radiochemical equipment for this radiochemistry. Consequently, to streamline new methodologies, and make them widely available, new radiochemical equipment is needed. A possible approach to attack the problem could be to develop radiosynthesis equipment with a higher flexibility. A fundamental question is if microscale technology (microfluidic or microreactor) can provide a breakthrough in radiochemistry? Its compact design, flexible attributes, and its suitability for automation make microscale technology an ideal platform for performing the rapid radiolabeling reactions required for PET. So far, efforts made to adapt microscale technology for PET radiolabeling purposes have focused on proof-of-principle studies and to illustrate the advantages associated with the technology and significant further development is needed for the technology to reach its full potential. Although the authors recognize the importance of microreactor technologies, other technical approaches towards the development of more flexible radiochemical synthesis equipment are equally attractive at this point. Regardless of which direction is taken in the future, we firmly believe that a stronger collaboration between radiochemists and technical engineers is vital for succeeding in the development of the next generation of PET radiochemistry equipment.

Finally, radiochemistry is the foundation for PET imaging. By broadening the spectrum of radiochemical reactions within clinical PET radiochemistry, radiochemists will not only be able to increase the number of compounds that can be labeled with carbon-11 but also provide an increased opportunity to label a given compound in different positions.
